# A modified score to identify and discriminate neuropathic pain: a study on the German version of the neuropathic pain symptom inventory (NPSI)

**DOI:** 10.1186/1471-2377-11-104

**Published:** 2011-08-23

**Authors:** Claudia Sommer, Helmut Richter, Jan P Rogausch, Jule Frettlöh, Margitta Lungenhausen, Christoph Maier

**Affiliations:** 1 Department of Neurology, University of Würzburg, Germany; 2 Department of Pain Management, BGliches Universitätsklinikum Bergmannsheil, Ruhr University Bochum, Germany

## Abstract

**Background:**

Neuropathic pain must be correctly diagnosed for optimal treatment. The questionnaire named Neuropathic Pain Symptom Inventory (NPSI) was developed in its original French version to evaluate the different symptoms of neuropathic pain. We hypothesized that the NPSI might also be used to differentiate neuropathic from non-neuropathic pain.

**Methods:**

We translated the NPSI into German using a standard forward-backward translation and administered it in a case-control design to patients with neuropathic (n = 68) and non-neuropathic pain (headache and osteoarthritis, n = 169) to validate it and to analyze its discriminant properties, its sensitivity to change, and to detect neuropathic pain subgroups with distinct profiles.

**Results:**

Using a sum score (the NPSI-G score), we found sensitivity to change (r between 0.37 and 0.5 for pain items of the graded chronic pain scale) and could distinguish between neuropathic and other pain on a group basis, but not for individual patients. Post hoc development of a discriminant score with optimized diagnostic properties to distinguish neuropathic pain from non-neuropathic pain resulted in an instrument with high sensitivity (91%) and acceptable specificity (70%). We detected six different pain profiles in the patient group with neuropathic pain; three profiles were found to be distinct.

**Conclusions:**

The NPSI-G potentially combines the properties of a diagnostic tool and an instrument to identify subtypes of neuropathic pain.

## Background

Neuropathic pain must be correctly diagnosed for optimal treatment. Several screening tools have been developed to differentiate neuropathic from non-neuropathic pain [1]. These include the Leeds Assessment of Neuropathic Symptoms and Signs (LANSS [2]), the Neuropathic Pain Questionnaire [3], the DN4 [4], the painDETECT questionnaire [5], and the ID pain [6]. Another goal of pain questionnaires is the use of effective pain descriptors to identify subgroups of patients that may benefit from specific therapies. Such questionnaires are the Neuropathic Pain Scale (NPS) [7] and the Neuropathic Pain Symptom Inventory (NPSI) [8]. The NPSI has psychometric properties which suggest that it may be used to characterize subgroups of neuropathic pain patients and verify whether they respond differentially to treatment [8]. It has good construct validity, high test-retest reliability, and is sensitive to change. It has been translated into several languages and used in clinical trials [9,10]; see additional file 1 for its (non-validated) English translation by the original authors. Given the good psychometric properties of the NPSI, we constructed and validated a German version, the NPSI-G with the aims to analyze its sensitivity to change and to identify distinct subgroups among patients with neuropathic pain. We hypothesized that the NPSI might also be used to differentiate neuropathic from non-neuropathic pain, a distinction which was not tested in the original study. We collected data from 237 participants with confirmed neuropathic pain, and with two frequently encountered non-neuropathic chronic pain syndromes, osteoarthritis, or headache. To enhance the separation qualities of the NPSI-G for neuropathic and non-neuropathic pain, we used discriminant analysis to construct a weighted sum score of the variables.

## Methods

### Patients

Patients with neuropathic pain and headache were recruited from the Department of Neurology, University of Würzburg and the Department of Pain Management (Bergmannsheil), Ruhr University, Bochum. Patients with osteoarthritis related pain were recruited from the Department of Orthopedics (König-Ludwig-Haus), University of Würzburg and the Department of Orthopedics (St. Josefs Klinikum), Ruhr University, Bochum. A diagnosis of neuropathic pain was confirmed when associated with a definite neurological cause as determined during routine in- or outpatient management of the patient. In addition, it was required that the pattern of pain distribution was characteristic of the respective diagnosis. Headache was diagnosed using the International Headache Society criteria for tension type headache and migraine [11]. Only patients with a headache present at the time of visit were included. Headache was chosen in order to have a pain condition with a very different distribution from that in most patients with neuropathic pain. Osteoarthritis was diagnosed based on criteria provided by the American College of Rheumatology (http://www.rheumatology.org). Osteoarthritis was chosen to include a condition that (like in most patients with neuropathic pain in our sample) entails pain in the extremities. Exclusion criteria were coexistence of several conditions possibly causing pain, severe depression, chronic alcoholism or substance abuse, and an inability to understand the questionnaire. Patients with neuropathic pain received current symptomatic treatment, including analgesic drugs, when admitted or seen as outpatients.

### NPSI Translation into German and adaptation of the NPSI for patients with headache and osteoarthritis

Using the original French NPSI, we used a standard forward-back translation to develop the German equivalent. First, two translators who were native French speakers and fluent in German (one with, one without medical knowledge) translated the questionnaire into German. A reconciled language version was developed using these two forward translations. A third professional translator (a native German speaker fluent in French) produced the back-translation. The final version was evaluated by three independent medical professionals fluent in both French and German usage. Discrepancies in wording were analyzed and when required, resolutions were obtained by consensus. A diagram depicting pain attacks and episodes of continuous pain was inserted to facilitate patient comprehension. For quality control, patients were instructed to indicate areas of pain on an additional diagram (see additional file 2 for a full version of the NPSI-G). Patients with headache or osteoarthritis related pain were additionally required to complete a short questionnaire that addressed aspects of their headache (questions were designed to differentiate between tension headaches and migraines) or, their joint pain (location of pain sites including potentially related sites).

### Study design

The study was approved by the Ethics Committees of the Universities of Bochum and Würzburg. After providing informed consent, the patients were required to complete four questionnaires: the NPSI-G, the German version of the NPS (NPS-D), the graded chronic pain scale (GCPS, [12]), and the German version of the Center for Epidemiologic Studies Depression Scale (CES-D) [13]. Patients diagnosed with neuropathic pain were instructed to complete a 2nd NPSI-G as well as the patient global impression of change (PGIC) scale [14] 24 hours later. The entire set of questionnaires was repeated at 4 weeks. Since most of these patients were inpatients, their 24 hour questionnaire was readily distributed and collected. Outpatients with neuropathic pain were given both the 24 hour and 4 week questionnaires with dates for completion clearly marked and prepaid postal envelopes addressed to the investigators. Patients who did not return the questionnaires at the specified time intervals received telephone reminders. During the 4-week period, patients underwent medical treatment as required. Patients with headache (mostly outpatients) or osteoarthritis pain (mostly inpatients) received all of the above questionnaires only once and were instructed to complete their questionnaires while at the hospital.

Depressive symptoms were assessed using the CES-D questionnaire. Scores above 23 points were considered indicative of depressive states. Pain intensities were graded using the 11-point numerical scales (0 - 10) of the GCPS.

### Sample description and comparison of the diagnostic groups

Sample description included diagnostic data, age, gender, depression score (CES-D) and general pain characteristics (GCPS scores) for each diagnostic group.

### Construction of a score for assessing neuropathic pain

A sum score (NPSI-G score) for assessing the severity of neuropathic pain was calculated using the sum score methodology of our reference study [8]. A possible positive contribution of the categorical items Q4 (frequency of spontaneous pain) and Q7 (number of pain attacks) to the score was examined by transforming them into dichotomous variables and post hoc adding weighted variable scores to the sum score. Then we looked for improvement by comparing the validity measures of the original and the expanded score. Optimized transformation rules and weight-coefficients were generated from an algorithm which tested possible improvements of the correlations in the validity calculations.

### Reliability and validity of the NPSI-D

For the group of patients with neuropathic pain, we assessed the test-retest reliability of the NPSI-G and the NPSIG score and analyzed its convergent and divergent validities. The test-retest reliability of the interval-scaled items and the NPSI-G score was assessed by calculating intra-class correlations (ICC, two way random model) for all items included in the initial measurements and the 24-hour follow-up. Since there are no validated instruments available in German comparable to the NPSI, we used two widely employed comparators for convergent validity; the German version of the NPS [7] (NPS-D) and those items from the GCPS that examine intensity of pain (current, average and maximal pain assessments over the 4 week interval). Divergent validity was measured by calculating the Pearson correlations with the CES-D scores. We then compared the reliability of the NPSI-G items and the NPSI-G score with the French reference study.

### Sensitivity to change

The Pearson correlations between the 4-week change of the NPSI-G score and the changes of current, average, and maximum pain in the GCPS, the PGIC-score, and the change of the CES-D-score were calculated in patients with neuropathic pain, whereby the CES-D was used as a control measure for divergent characteristics. Correlations were considered low, medium or high as they reached levels of r = 0.1, r = 0.3, or r = 0.5 respectively [15]. To conclude that there was sensitivity to change, we would expect at least medium correlations with the changes of pain intensity in the CGPS (r ≥ 0.3). As the PGIC score is a gross estimate of changes in pain intensity, it was only considered as an additional control instrument. A low to medium correlation (r ≥ 0.2) was judged sufficient to support the hypothesis that the NPSI-G score is sensitive to change. Low correlations with the ASD-score (r < 0.3) would support divergent validity.

### Evaluation of the discriminant properties of the NPSI-G and the NPSI-G score

Responses to the NPSI-G items were compared between the diagnostic groups by analyzing the mean values and the frequencies of positive item responses (values > 0), indicating the occurrence of the different qualities of pain for the 10 interval scaled items Q1 - Q3, Q5 - Q6, Q8 - Q12. Mean values of items and sum score were calculated and compared between diagnostic groups. The distribution of the NPSI-G score within the groups was analyzed using box plots. The correlation of the NPSI-G score with age, sex, pain intensity in the CGPS, and the CES-D score was calculated by Pearson correlations to control for a possible bias caused by between-group differences of these parameters. We then examined the diagnostic power of the NPSI-G score using a receiver operating characteristic (ROC) diagram [16]. The ROC diagram shows the possible combinations of sensitivity and specificity that can be achieved for a given score. The non-neuropathic group was formed by combining the osteoarthritis and the headache patients.

### Development and analysis of a diagnostic tool based on the NPSI-G

To develop a tool that would separate patients with neuropathic pain from the other groups, we calculated a discriminant score (NPSI-G-dis) using discriminant analysis. This weighted sum score of the variables was constructed using specific coefficients for each variable to allow optimal separation of the different diagnostic groups. We used all variables from the NPSI-G including the two categorical variables Q4 and Q7, which were transformed into dichotomous variables similar to the construction described in the definition of the NPSI-G score, but without weight coefficients which were dispensable in this context. In this case an optimal ROC-diagram was the criterion for an appropriate transformation.

### Analysis of different pain profiles in patients with neuropathic pain

Using a cluster analysis (hierarchical Ward analysis [17] with follow up k-means analysis) based on the ten interval items of the NPSI-G, we looked for subgroups with different pain profiles within the neuropathic pain group. Profiles were compared by multivariate variance analysis (MANOVA; Wilks-Lambda used as test statistic) and differences between the item scores within each cluster by variance analysis for dependent measures. An analysis of current and maximal pain levels from the GCPS was done to evaluate whether clusters were based on pain severity only. The distribution of pain intensities was examined by box plots; mean values for different clusters were compared by t-tests.

### Test statistics and measurements of coherence

In addition to the already described methods, we used the following statistical tests: Frequencies of item responses were compared using Chi-square tests. Comparison of group means (of the different diagnostic groups or of groups defined by the results of the cluster analysis) was done by t-tests in the case of two groups and by ANOVAs with post hoc tests (Sidak resp. Dunnett T-3 adjustments for multiple testing) when more than two independent groups were involved [18,19]. To analyze coherence of item responses, we calculated Pearson correlations. Compensation for multiple comparisons was done by Bonferroni adjustments [20].

## Results

### Sample description and comparison of the diagnostic groups

Out of 255 patients screened, we recruited 241 participants who fulfilled the inclusion criteria. Four patients (n = 2 with osteoarthritis and headache each) were later excluded from analysis because of failure to complete the NPSI-questionnaire. The study cohort consisted of the remaining 237 patients. Patient data are provided in Table 1. The mean age of patients with neuropathic pain and with osteoarthritis was nearly the same (p = 0.88) whereas headache patients were younger (p < 0.001 for either comparison). There were fewer women in the neuropathic pain group (p < 0.05 for the overall comparison). Pain intensities from the GCPS differed between the three diagnostic groups (see Table 1, p < 0.05 for all items in the overall tests). Fewer patients with osteoarthritis had depressive symptoms than patients in the other diagnostic groups (p < 0.01).

**Table 1:** Demographic and clinical data of the study participants

Clinical and demographic data		Diagnosis		
	NP	OA	H	all
N	68	93	76	237
mean age	59.1 ± 13.0	60.5 ± 12.4	49.7 ± 14.7	56.6 ± 14.1
(range)	(26-85)	(22-83)	(20-83)	(20-85)
Sex (%women)	35.3	52.2	55.4	48.3
mean pain intensity on a numeric 0-10 - pain scale (SD)				
current pain	4.9 (2.8)	5.5 (2.8)	4.1 (3.0)	4.9 (2.9)
maximal pain intensity in the past 4 weeks	8.1 (1.8)	6.8 (2.6)	7.8 (2.2)	7.5 (2.3)
average pain intensity in the past 4 weeks	6.1 (2.1)	6.2 (2.6)	5.3 (2.3)	5.9 (2.4)

**Diagnosis**			**N (%)**	
		**CES-D≥23**	**CES-D <23**	**all**

**NP**		25 (42)	34 (58)	**68 (29)**
	central pain			8 (11)
	nerve injury pain			10 (15)
	peripheral neuropathy			50 (74)
**OA**		15 (18)	70 (82)	**93 (39)**
	gonarthritis			39 (42)
	coxarthritis			26 (28)
	others			28 (30)
**H**		30 (41)	44 (59)	**76 (32)**
	migraine			58 (76)
	tension headache			18 (24)
**all**		70 (30)	148 (70)	**237 (100)**

NP: Neuropathic pain; OA: osteoarthritis; H: headache; depr: depressive

### The NPSI-G score

Cronbach's alpha for the 10 interval items (α = 0.75) allowed accepting a one dimensional latent construct and thus calculating a sum score [21,22], the NPSI-G score. This calculation was different from the algorithm used in the reference study, where the authors had extracted a 5-dimensional factor solution leading to sub-scores for each dimension, which were then added to obtain an overall sum score. The inclusion of the transformed categorical items Q4 and Q7 did not lead to remarkable improvements of the correlations shown in Table 2; in most cases, correlations were even lower. The best result was a gain of 0.02 points for the correlation with average pain. Thus, the original NPSI-G score was retained with the exclusion of items Q4 and Q7.

**Table 2:** Intraclass correlation coefficients (ICC) for each item and for the NPSI sum scores based on baseline and second measurement (after 24 h in the current study and after 3 h in the reference study)

	Current study	Reference study
Burning	0.66	0.95
Squeezing	0.82	0.91
Pressure	0.75	0.98
Electric shocks	0.95	0.90
Stabbing	0.79	0.92
Evoked by brushing	0.70	0.97
Evoked by pressure	0.72	0.95
Evoked by cold stimuli	0.79	0.88
Pins and needles	0.84	0.98
Tingling	0.84	0.87
**Sum score**	**0.89**	**0.94**

### Reliability and validity

The test-retest reliabilities for a 24 h time interval are shown in Table 3. Only four items obtained an ICC higher than 0.80, with one item (burning) having an ICC of only 0.66. Despite this restricted stability on the item level, the NPSI-G score was highly reliable (ICC = 0.89) which is comparable to the sum score of the reference study (ICC = 0.94).

**Table 3:** Convergent and divergent validities (Pearson correlations) for patients with neuropathic pain

	correlation with NPSI-D score
NPS-D score	0.87
current pain	0.69
maximal pain (past 4 weeks)	0.42
average pain (past 4 weeks)	0.41
CES-D-score	0.14

Construct validity information is given in Table 3. The correlation of the NPSI-G score with the NPS-D score was high. As expected, the correlation with current pain intensity in the GCPS was also high in contrast to medium correlations with maximum pain and average pain in the past 4 weeks. The correlation with the depression-score was low and supported divergent validity.

### Sensitivity to change

The Pearson correlations for a 4-week change in the NPSI-G score, a parallel change in the GCPS and the PGIC are shown in Table 4. As expected, we found medium to high correlations with changes of current pain and average pain (r-values nearly 0.5) and a medium correlation with maximal pain. Correlations with the PGIC were low to medium (nearly 0.3). Divergent validity was shown by the low correlation with changes in the CES-D.

**Table 4:** Correlation of changes in a 4-week period in patients with neuropathic pain (Pearson correlations)

	NPSI-D
current pain	0.46
maximal pain (past 4 weeks)	0.39
average pain (past 4 weeks)	0.49
PGIC	0.28
CES-D	0.23

### Evaluation of the discriminant properties of the NPSI-G

The pain drawings confirmed that the pain the patients described was directly related to the syndrome they were initially diagnosed with: headache patients localized their pain to the head, the osteoarthritis patients to the respective joints, and the patients with neuropathic pain to their feet or the area of their nerve lesion. Table 5 shows the characteristics of the item responses for the different diagnostic groups. A tendency for a higher number of positive answers was observed in the neuropathic pain group. Mean values for neuropathic pain patients however, only significantly exceeded those in the other diagnostic groups for the items "burning pain", "pins and needles", and "tingling".

**Table 5:** Frequency of positive NPSI item responses

	NP	OA	p (NP vs. OA)	H	p (NP vs. H)	reference study^#^
**Burning**	73.4	31.9	< 0.001*	30.8	< 0.001*	70.5
**Squeezing**	50.8	24.3	0.001*	53.1	0.859	63.4
**Pressure**	64.4	67.0	0.857	87.5	0.003*	60.6
**Electric shocks**	42.6	21.9	0.010*	42.2	1.000	61.2
**Stabbing**	68.8	68.9	1.000	61.6	0.468	60.1
**Evoked by brushing**	31.1	36.4	0.596	15.9	0.040*	68.5
**Evoked by pressure**	62.5	62.9	1.000	38.0	0.006*	67.5
**Evoked by cold**	29.5	17.9	0.114	27.7	0.849	42.5
**Pins and needles**	77.7	26.1	< 0.001*	35.6	< 0.001*	63.0
**Tingling**	52.3	26.4	< 0.001*	30.5	0.014*	66.4

**Means for item and sum** **scores (SD)**						
	**NP**	**OA**	**p** **(NP vs. OA)**	**H**	**p** **(NP vs. H)**	**all**

**Burning**	4.7 (3.4)	2.0 (3.3)	< 0.001*	1.8 (2.8)	< 0.001*	2.8 (3.4)
**Squeezing**	3.3 (3.6)	1.4 (2.7)	< 0.001*	3.2 (3.4)	0.804	2.5 (3.3)
**Pressure**	3.7 (3.2)	4.0 (3.4)	0.697	5.4 (3.2)	0.004*	4.4 (3.4)
**Electric shocks**	2.6 (3.4)	1.4 (2.9)	0.026*	2.1 (3.1)	0.396	2.0 (3.1)
**Stabbing**	4.5 (3.6)	4.4 (3.5)	0.924	4.1 (3.8)	0.543	4.3 (3.6)
**Evoked by brushing**	1.7 (3.7)	2.1 (3.6)	0.401	0.9 (3.3)	0.110	1.6 (3.6)
**Evoked by pressure**	4.0 (3.7)	4.1 (3.6)	0.957	2.2 (3.3)	0.003*	3.5 (3.6)
**Evoked by cold**	1.7 (2.9)	1.0 (2.4)	0.111	1.4 (2.6)	0.460	1.3 (2.6)
**Pins and needles**	4.4 (3.2)	1.2 (2.3)	< 0.001*	1.5 (2.4)	< 0.001*	2.2 (2.9)
**Tingling**	3.3 (3.5)	1.3 (2.4)	< 0.001*	1.3 (2.4)	< 0.001*	1.9 (2.9)
	**33.2**	**22.5**		**22.5**		
**Sum score**	**(19.5)**	**(14.1)**	< 0.001*	**(14.1)**	< 0.001*	

NP: Neuropathic pain; OA: osteoarthritis; H: headache; depr: depressive *: p < 0.05

#: frequencies for neuropathic pain patients in the French reference study [8].

**Figure 1:**
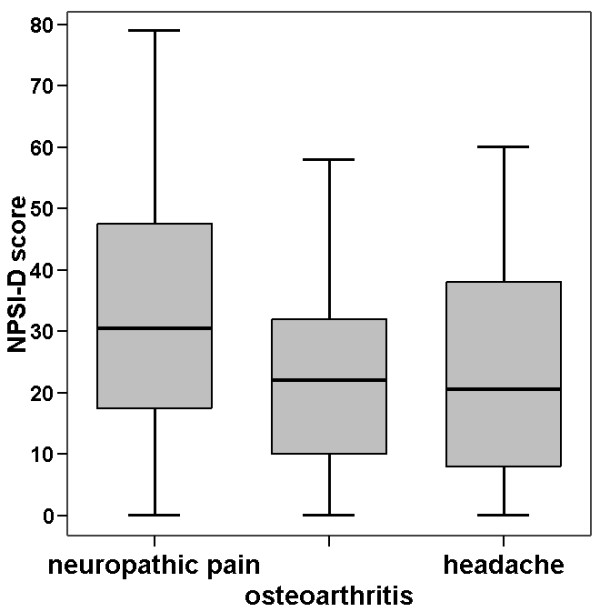
**NPSI-G score for different syndromes**. The box plots show medians and ranges, the whiskers mark the first and the fourth quartiles. Outliers with a distance from the median that exceeds three times the box length are excluded. Differences between neuropathic pain and the other diagnoses are statistically significant (both p < 0.05).

**Figure 2:**
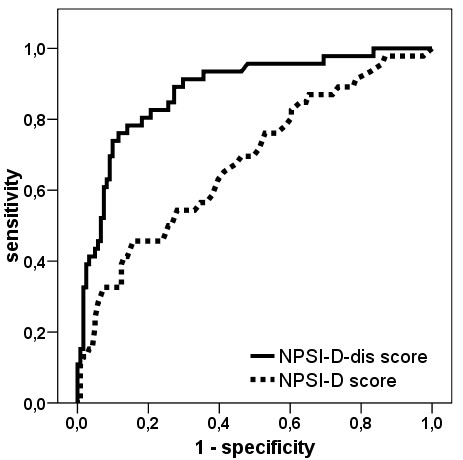
**ROC-curves for the discriminant score NPSI-G-dis and the NPSI-G score**. The graphs show the possible combinations of sensitivity/specificity values that can be achieved by varying the cut-off values for each score.

When we calculated the NPSI-G scores for the different diagnostic groups, the mean value for neuropathic pain patients was significantly higher than that of the comparison groups (p < 0.05 for both comparisons with neuropathic pain, adjusted α). There was no significant difference between the two comparison groups (p > 0.98). In spite of these findings, the box plots in Figure 1 demonstrate a wide overlap which indicates a poor separation quality of the NPSI-G score. This was confirmed by the ROC diagram (Figure 2), which demonstrated only unacceptable combinations of sensitivity/specificity.

### Construction and analysis of a diagnostic tool based on the NPSI-G

Since the separation quality of the NPSI-G score for neuropathic versus non-neuropathic pain was not satisfactory, we used discriminant analysis to construct an improved score for the separation of neuropathic and non-neuropathic pain. The optimal transformations of the categorical items 4 and 7 into dichotomous scores are: Q4_d = 0/1 if Q 4 ≥ 4/<4; Q7_d = 0/1 if Q7 ≥2/<2 (allocation of higher scores to lower class numbers corresponds to the order of classes in the questionnaire, smaller numbers indicate higher numbers of complaints).

The resulting post-hoc discriminant score (NPSI-G-dis) is calculated by the coefficients given in Table 6, using them as weight factors for the item scores.

**Table 6:** Discrimination-coefficients for the discrimination score

	Item	Coefficient
Q1:	burning	1.8
Q2:	squeezing	1.0
Q3:	pressure	-0.6
Q4_d:	spontaneous pain (dichot.)*	0.4
Q5:	electric shocks	-0.3
Q6:	stabbing	-0.7
Q7_d:	pain attacks (dichot.)*	-8.0
Q8:	evoked by brushing	-0.5
Q9:	evoked by pressure	0.5
Q10:	evoked by cold stimuli	-0.6
Q11:	pins and needles	3.7
Q12:	tingling	-1.3
constant value		50.0

* Q4 and Q7 are dichotomized by setting Q4_d = 1 if Q4<4

resp. Q7_d = 1 if Q7<2 and appointing 0 in the other cases

**Figure 3:**
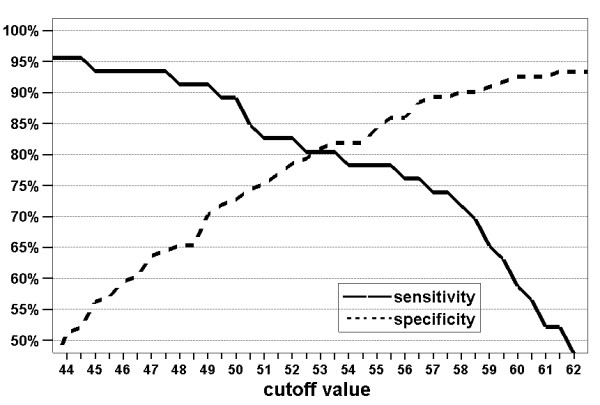
**Sensitivity and specificity achieved for the discriminant score as a function of the chosen cut-off values**.

To obtain a flexible diagnostic instrument, appropriate cut-off values need to be chosen. Figure 3 shows the possible combinations of sensitivity and specificity for the NPSI-G-dis. Some possible value pairs for sensitivity/specificity are (cut-off values in brackets): 91%/70% (49.0), 80%/82% (53.5) or 72%/90% (58.0). The discriminant score led to a clear improvement of diagnostic quality compared to the sum score which can be seen in the ROC diagram (Figure 2). The correlations of the NPSI-G-dis with group parameters were low to negligible: r = 0.11/0.05/0.15 for current pain/average pain/maximal pain from the GCPS, p = 0.17/0.52/0.053 and r = 0.11/0.22/0.21 for age/gender/depression, p = 0.16/0.003/0.005.

### Pain profiles of neuropathic pain patients

**Figure 4:**
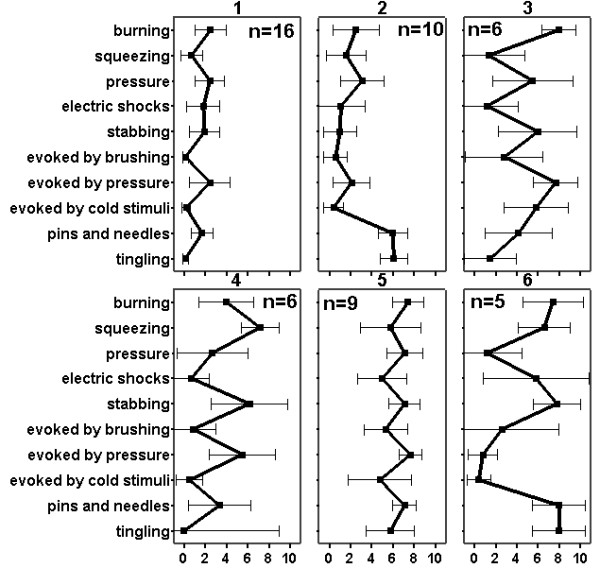
**Results of a cluster analysis for patients with neuropathic pain**. The graph shows mean values and 95% confidential intervals of item responses for a 6-cluster solution. The profiles of clusters 1, 2 and 5 are mutually different with statistical significance (p < 0.05 after adjustment for multiple comparisons). x-axis: numerical rating scale (NRS), pain intensity on a 11-point numerical rating scale (0 - 10).

**Figure 5:**
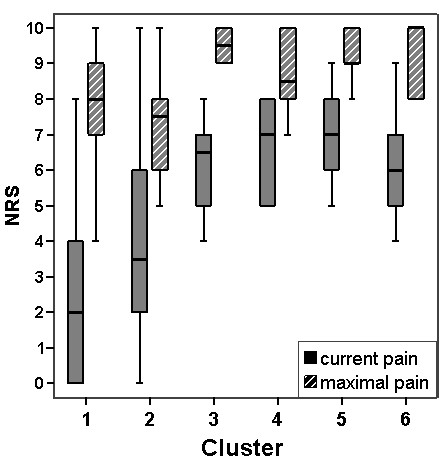
**Box plots for current pain and maximal pain**. The box plots show current pain and maximal pain in the past 4 weeks in each of the 6 subgroups of neuropathic pain patients found by cluster analysis. NRS: pain intensity on a 11-point numerical rating scale.

The cluster analysis led to a six-cluster solution (Figure 4). Comparing the profiles by multivariate variance analysis led to a significant result for the overall analysis (p < 0.001). Using pairwise testing (with Bonferroni adjustment for 15 comparisons, α = 0.05 as target), the profile of cluster 1 was different from all other clusters. Furthermore, the profile of cluster 2 was different from the clusters 4 and 5. Three of the profiles, the clusters 1, 2, and 5 (n_1 _= 16, n_2 _= 10, n_5 _= 9), were mutually different with statistical significance. The differences between cluster 1 and 2 or between cluster 2 and 5 were due to different shapes and not to different levels alone. Clusters 1 and 2 were different for the items "pins and needles" and "tingling" (p < 0.001 for both comparisons) whereas all other differences may be neglected (p > 0.27 for all cases). A comparison of cluster 2 and 5 led to the opposite result, with no difference in the items "pins and needles" and "tingling" (p = 0.180 resp. p = 0.774) but significant differences for all other items (p < 0.015 for each case). Cluster 1 and 5 were similar in shape but with different levels of values. To control for the influence of global pain intensity on the differences between clusters, we show in Figure 5 the distribution of the pain intensities according to the CGPS in each cluster. For example there were no differences between the pain intensities of cluster 1 and 2 thus indicating that differences in profiles cannot be attributed to differences in pain intensities alone.

## Discussion

### Reliability and validity of the NPSI and comparison of the item characteristics to the French original and to transnational results

The NPSI-G score demonstrated a high test-retest reliability and convergent as well as divergent construct validity when applied to neuropathic pain patients. This allows us to conclude that the NPSI-G is a useful and reliable instrument for assessing the severity of neuropathic pain. However, since our patient population was biased toward neuropathic pain of peripheral origin, the NPSI-G will have to be validated in a separate sample including also patients with other neuropathic pain syndromes, e.g. with central pain. Nearly all properties of the NPSI-G score were comparable to the original French results. In contrast to the reference study, test-retest-reliability on a per item basis did not reach acceptable values in all cases. This might possibly be attributed to the different time lags, which were 24 h in the current as opposed to 3 h in the reference study. The possibility of different connotations of some items in the respective languages as another possibility for divergent results has recently been discussed for several non-validated translations of the NPSI [9]. For neuropathic pain patients, the responder rates for individual items were more variable than in the French study (see Table 5). Ranking the items according to responder rates of neuropathic pain patients resulted in pins and needles as the most frequently endorsed item, followed by burning, stabbing and pressure/evoked by pressure. The other items clearly achieved lower percentages. The differences in item ranking between the German and the French study may be caused by the composition of the neuropathic pain group. While our group consisted of a majority of patients with peripheral neuropathy, there were more patients with nerve trauma and with central pain in the French study which may explain the higher prevalence of brush evoked and electric shock like pain in their sample. A recent multinational study [9] found that across different cultures and languages (German not included), the ranking was the same for the items with the highest frequency of positive answers: "pins and needles", "burning" and "electric shock like". This is similar to our results, except for the third item which was "stabbing" in our study. However, the diagnostic composition within groups from the participating countries varied greatly, and numbers of participants from each country were small such that direct comparisons with the German and the French cohort are difficult, not excluding further limitations inherent in cross-cultural comparisons [22].

The correlation with data from the CGPS for current pain was high, for pain in the last 4 weeks it was only medium high (see Table 3). This result was predictable because the NPSI-G asks for current pain symptoms only. In the reference study, there was a correlation of ρ = 0.6 for the NPSI with general pain, comparable to our result for current pain (see Table 3). The low correlation with a depression scale was equivalent to the French study (ρ = 0.32).

### Sensitivity to change

The NPSI-G score is sensitive to change and thus allows measurement of treatment efficacy. As expected, there was only a weak correlation between the patients' assessment of change using the PGIC at 4 weeks and the NPSI-G score at 4 weeks. In this analysis, the patients' assessment of change over a relatively long period of time was compared with the difference between two assessments of current symptoms 4 weeks apart. From a psychological point of view, this analysis would not be expected to demonstrate strongly correlated results [23].

### Discriminant properties of the NPSI-G

The group of patients with neuropathic pain had a typical profile of pain descriptors that differed significantly from the control groups with osteoarthritis and headache (see Table 5). Similar group differences were achieved using the NPSI-G score. The NPSI-G score however, lacks discriminant power when used for individual patients, as demonstrated in Figure 1 and in the ROC diagram (Figure 2).

### The NPSI-G as a tool for diagnosing neuropathic pain

The post hoc construction of a discriminant score based on the algorithm of discriminant analysis led to clearly enhanced separation qualities. For example, a high sensitivity of 91% combined with a tolerable specificity of 70% or a combination of 72% sensitivity and 90% specificity were possible. The quality of this diagnostic instrument is slightly inferior to the diagnostic tool based on the French DN4 questionnaire (sensitivity 82.9%, specificity 89.9%) [4], which was constructed with a selection of items from the NPSI and some additional items.

### The NPSI-G as a tool for analyzing distinct neuropathic pain profiles

The cluster analysis revealed six clusters with different patterns. Three of them (clusters 1, 2, and 5 in Figure 4) showed differences in pairwise comparisons that were statistically significant in multivariate testing. Comparable patterns but different levels of pain intensity were found in two clusters (clusters 1 and 5), whereas cluster 2 demonstrated a different shape mainly caused by the dysesthesia items. Overall, the clusters do not merely represent distinctive pain intensities as shown by the box plots in Figure 5, and the different profiles cannot be explained by different pain levels alone. For example, clusters 3 to 6 are very similar in pain levels yet show very different profiles. Most of these differences are statistically significant in simple comparisons. Whether the clusters have a clinical meaning regarding pathophysiology or response to treatment cannot be answered by our study. Additional studies with larger numbers of participants are required to confirm our results. Furthermore, we will need to examine whether the clusters represent different underlying pain mechanisms.

### Limitations of the study

There are several limitations to our study. Limitations in assessing test-retest reliability must be considered as even a latency period of 24 h may be too short to guarantee independent measurements.

It was difficult to assess reliability because other procedures (for example parallel measuring) were not possible, and with any latency longer than 24 h, influences of treatment or spontaneous fluctuations in pain characteristics that would disturb identical conditions for the responses to the items would have to be expected. Given the high number of statistical tests, some spuriously random results cannot be excluded with certainty. The discriminant function was optimized for the given data, and we did not crossvalidate the results. Crossvalidation was not possible because of the relatively small sample size. Our results indicate that a follow-up study to achieve this aim might be worthwhile. Differences in group parameters (depressiveness, age, gender, pain intensity) may have caused some biases when analyzing group differences. This mainly concerns responses to single items or the NPSI-G score. The correlations of this score (calculated for the whole sample) with age and gender are low, but are considerable for the pain variables (about 0.5) and for depressiveness (> 0.3). The discriminant score NPSI-G-dis was only marginally influenced by these parameters, with only low correlations.

## Conclusions

The NPSI-G is the first questionnaire that is capable of discriminating subtypes of neuropathic pain and provides additional information as a diagnostic tool. Using specifically constructed scores, the NPSI-G can distinguish neuropathic from non-neuropathic pain with high sensitivity and acceptable specificity. This combination of properties suggests that it may be ideally suited for use in clinical trials. Further studies with larger numbers of participants are required to confirm our findings.

## Competing interests

The authors declare that they have no competing interests.

## Authors' contributions

CS participated in the design of the study, in data collection and writing of the manuscript. HR performed the statistical analysis and participated in writing of the manuscript. JPR participated in data collection and data analysis. JF participated in the design of the study and data analysis. ML participated in data collection and data analysis. CM participated in the design of the study, statistical analysis, and writing of the manuscript. All authors read and approved the final manuscript.

## Pre-publication history

The pre-publication history for this paper can be accessed here:

http://www.biomedcentral.com/1471-2377/11/104/prepub

## Supplementary Material

Additional file 1 **English translation of the original NPSI by Bouhassira et al. (2004)**. This is not a not a back translated and validated version of the NPSI, do not use! The English translation is only given for the benefit of those readers who do not read German or French. Click here for file

Additional file 2 **Full version of the NPSI-G as it was used in the study**. Click here for file
