# Supplementary material for: A modified score to identify and discriminate neuropathic pain: a study on the German version of the neuropathic pain symptom inventory (NPSI)
Source: BMC Neurol. 2011 Aug 23;11:104. doi: 10.1186/1471-2377-11-104 (PMC3180265; doi:10.1186/1471-2377-11-104)
Supplement: Additional file 1 — English translation of the original NPSI by Bouhassira et al. (2004). This is not a not a back translated and validated version of the NPSI, do not use! The English translation is only given for the benefit of those readers who do not read German or French. [file 1471-2377-11-104-S1.DOC]

# English translation of the original NPSI by Bouhassira et al. (2004). This is not a not a back translated and validated version of the NPSI, do not use! The English translation is only given for the benefit of those readers who do not read German or French

# NEUROPATHIC PAIN SYMPTOM INVENTORY

**You are suffering from pains due to injury or disease of the nervous system. These pains may be of several types. You may have spontaneous pain, that is pain in the absence of any stimulation, which may be long-lasting or occur as brief attacks. You may also have pain provoked or increased by brushing, pressure, contact with cold in the painful area. You may feel one or several types of pain. This questionnaire has been developed to help your doctor to better evaluate and treat the various types of pain you feel.**

*We wish to know if you feel spontaneous pain, that is pain without any stimulation. For each of the following questions, please select the number that best describes your* ***average spontaneous pain severity during the past 24 hours.*** *Select the number 0 if you have not felt such pain. (circle one number only)*

Q1/. Does your pain feel like burning ?

| No  burning | 0 | 1 | 2 | 3 | 4 | 5 | 6 | 7 | 8 | 9 | 10 | Worst  burning imaginable |
| --- | --- | --- | --- | --- | --- | --- | --- | --- | --- | --- | --- | --- |

Q2/. Does your pain feel like squeezing ?

| No  squeezing | 0 | 1 | 2 | 3 | 4 | 5 | 6 | 7 | 8 | 9 | 10 | Worst  squeezing imaginable |
| --- | --- | --- | --- | --- | --- | --- | --- | --- | --- | --- | --- | --- |

Q3/. Does your pain feel like pressure ?

| No  pressure | 0 | 1 | 2 | 3 | 4 | 5 | 6 | 7 | 8 | 9 | 10 | Worst  pressure imaginable |
| --- | --- | --- | --- | --- | --- | --- | --- | --- | --- | --- | --- | --- |

Q4/. **During the past 24 hours,** your spontaneous pain has been present :

*Select the response that best describes your case*

|  | Permanently  Between 8 and 12 hours  Between 4 and 7 hours  Between 1 and 3 hours  Less than 1 hour | /_/  /_/  /_/  /_/  /_/ |
| --- | --- | --- |

*We wish to know if you have brief attacks of pain. For each of the following questions, please select the number that best describes the* ***average severity of your painful attacks during the past 24 hours****. Select the number 0 if you have not felt such pain. (circle one number only)*

Q5/. Does your pain feel like electric shocks ?

| No electric shocks | 0 | 1 | 2 | 3 | 4 | 5 | 6 | 7 | 8 | 9 | 10 | Worst electric  shocks imaginable |
| --- | --- | --- | --- | --- | --- | --- | --- | --- | --- | --- | --- | --- |

Q6/. Does your pain feel like stabbing ?

| No  stabbing | 0 | 1 | 2 | 3 | 4 | 5 | 6 | 7 | 8 | 9 | 10 | Worst  stabbing imaginable |
| --- | --- | --- | --- | --- | --- | --- | --- | --- | --- | --- | --- | --- |

Q7/. **During the past 24 hours,** how many of these pain attacks have you had ?

*Select the response that best describes your case*

|  | More than 20  Between 11 and 20  Between 6 and 10  Between 1 and 5  No pain attack | /_/  /_/  /_/  /_/  /_/ |
| --- | --- | --- |

*We wish to know if you feel pains provoked or increased by brushing, pressure, contact with cold or warmth on the painful area. For each of the following questions, please select the number that best describes the* ***average severity of your provoked pains during the past 24 hours****. Select the number 0 if you have not felt such pain. (circle one number only)*

Q8/. Is your pain provoked or increased by brushing on the painful area ?

| No  pain | 0 | 1 | 2 | 3 | 4 | 5 | 6 | 7 | 8 | 9 | 10 | Worst  pain imaginable |
| --- | --- | --- | --- | --- | --- | --- | --- | --- | --- | --- | --- | --- |

Q9/. Is your pain provoked or increased by pressure on the painful area ?

| No  pain | 0 | 1 | 2 | 3 | 4 | 5 | 6 | 7 | 8 | 9 | 10 | Worst  pain imaginable |
| --- | --- | --- | --- | --- | --- | --- | --- | --- | --- | --- | --- | --- |

Q10/. Is your pain provoked or increased by **contact** with something cold on the painful area ?

| No  pain | 0 | 1 | 2 | 3 | 4 | 5 | 6 | 7 | 8 | 9 | 10 | Worst  pain imaginable |
| --- | --- | --- | --- | --- | --- | --- | --- | --- | --- | --- | --- | --- |

*We wish to know if you feel abnormal sensations* ***in the painful area****. For each of the following questions, please select the number that best describes the* ***average severity of your abnormal sensations during the past 24 hours****. Select the number 0 if your have not felt such sensation. (circle one number only)*

Q11/. Do you feel pins and needles ?

| No pins  & needles | 0 | 1 | 2 | 3 | 4 | 5 | 6 | 7 | 8 | 9 | 10 | Worst pins  & needles imaginable |
| --- | --- | --- | --- | --- | --- | --- | --- | --- | --- | --- | --- | --- |

Q12/. Do you feel tingling ?

| No  tingling | 0 | 1 | 2 | 3 | 4 | 5 | 6 | 7 | 8 | 9 | 10 | Worst  tingling imaginable |
| --- | --- | --- | --- | --- | --- | --- | --- | --- | --- | --- | --- | --- |
