# Supplementary material for: A modified score to identify and discriminate neuropathic pain: a study on the German version of the neuropathic pain symptom inventory (NPSI)
Source: BMC Neurol. 2011 Aug 23;11:104. doi: 10.1186/1471-2377-11-104 (PMC3180265; doi:10.1186/1471-2377-11-104)
Supplement: Additional file 2 — Full version of the NPSI-G as it was used in the study. [file 1471-2377-11-104-S2.DOC]

**FRAGEBOGEN NEUROPATHISCHE SCHMERZEN**

Datum: ……………..

Vorname: …………………………………… Name: ……………………………………….

Geschlecht: ……………………..

Alter: ……………………

Sie leiden an Schmerzen.

Möglicherweise haben Sie so genannte Spontanschmerzen, die ohne einen erkennbaren Auslöser auftreten. Diese sind entweder dauerhaft, d.h. ohne Unterbrechung ständig vorhanden, treten mit Unterbrechungen auf und/oder schwanken in ihrer Stärke. Zum besseren Verständnis haben wir versucht, dies anhand der folgenden Bilder deutlich zu machen:

| Dauerschmerzen  mit leichten Schwankungen | Dauerschmerzen  mit starken Schwankungen | Schmerzattacken (unterbrochener Schmerz) dazwischen schmerzfrei | Schmerzattacken (unterbrochener Schmerz) auch dazwischen Schmerzen |
| --- | --- | --- | --- |

Vielleicht haben Sie auch Schmerzen, die durch bestimmte äußere Auslöser (Berührung, Druck, Kälte) hervorgerufen werden können.

Bei einem Patienten können gleichzeitig mehrere Arten von Schmerzen bestehen.

Schmerzen sind für Patienten oft schwer zu schildern und Ärzte haben häufig Schwierigkeiten diese richtig zu verstehen. Deswegen werden Fragebögen entwickelt, die den Ärzten helfen sollen, die Schmerzen ihrer Patienten auch im Einzelnen zu erfassen. Dieses ist ein neuartiger Fragebogen, dessen Nützlichkeit wir testen wollen. Dazu benötigen wir Ihre Hilfe.

**Wo haben Sie Schmerzen ?**

Vorab möchten wir Sie fragen, wo überall Sie unter Schmerzen leiden.

Malen Sie bitte in den nachfolgenden Körperschemata ein, wo Sie überall Schmerzen haben.

Bitte kennzeichnen Sie das ganze Schmerzgebiet (durch Schraffierung mit Bleistift oder Kugelschreiber), damit wir wirklich wissen, wo Sie überall Schmerzen haben.


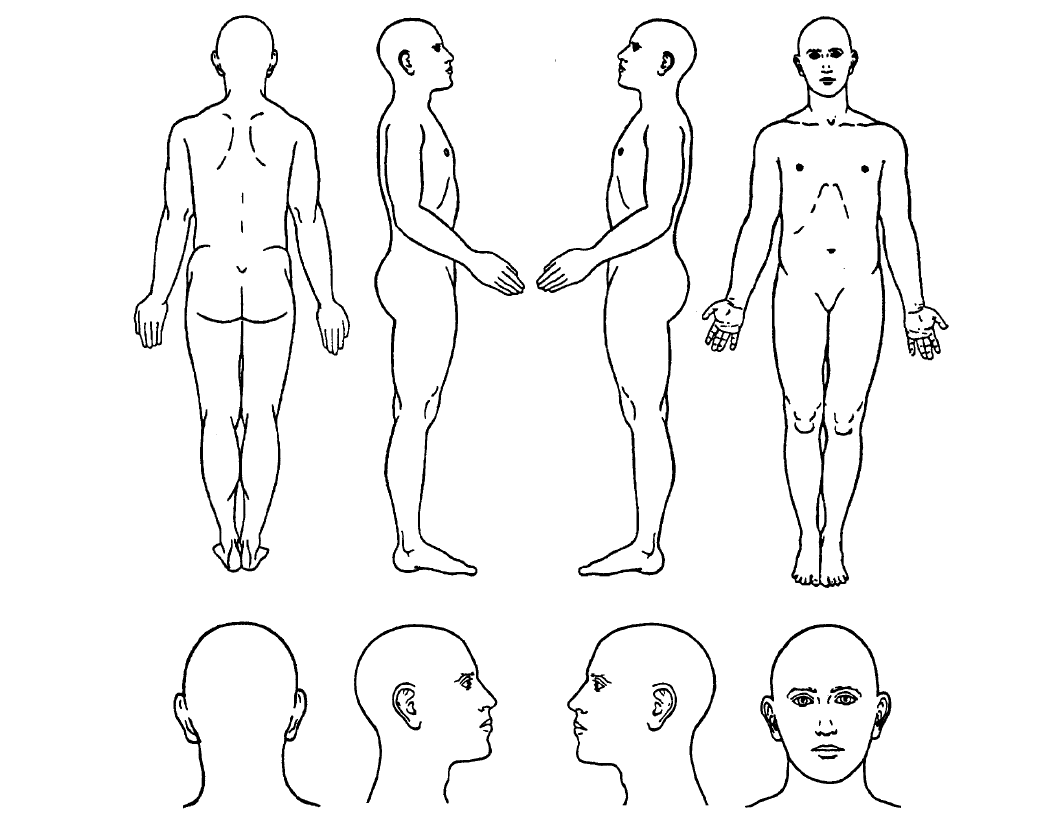


**Nehmen Sie Medikamente?**

Haben Sie in den letzten 24 Stunden Medikamente gegen Ihre Schmerzen genommen?

🞎 ja 🞎 nein

Welche Medikamente haben Sie **in den letzten 24 Stunden** gegen Ihre Schmerzen genommen?

| **Name des Medikaments** | **Wie oft haben Sie das Medikament genommen?** | **Wie viele Tabletten/**  **Tropfen /Zäpfchen haben Sie genommen?** |
| --- | --- | --- |
| z.B. Paracetamol 500 mg | 2 mal | jeweils 2 Tabletten |
|  |  |  |
|  |  |  |
|  |  |  |

Sie werden, wie Sie es vielleicht schon aus anderen Fragebogen kennen, nach der Stärke ihrer Beschwerden gefragt. Da man die Stärke von Beschwerden nicht einfach messen kann, verwenden wir hier eine Skala von 0 bis 10. 0 bedeutet dabei immer, dass Sie die entsprechenden Beschwerden nicht haben. 10 bedeutet, dass Sie die Beschwerden in der für Sie schlimmsten vorstellbaren Stärke haben.

**Spontanschmerzen**

Die ersten Fragen beziehen sich nur auf Spontanschmerzen, d.h. solche Schmerzen die ohne äußere Auslöser auftreten.

Haben Sie **Spontanschmerzen**, d. h. Schmerzen, die ohne äußeren Auslöser auftreten?

Bitte kreuzen Sie für jede der folgenden Fragen die Ziffer an, die am besten der **Stärke Ihrer Spontanschmerzen im Mittel über die letzten 24 Stunden entspricht**. Kreuzen Sie „0“ an, wenn Sie diese Art Schmerz nicht verspürt haben. (kreuzen Sie bitte immer nur eine Ziffer an)

Q1. Ist Ihr Schmerz brennend?

| kein Brennen | 0 | 1 | 2 | 3 | 4 | 5 | 6 | 7 | 8 | 9 | 10 | schlimmstes vorstellbares Brennen |
| --- | --- | --- | --- | --- | --- | --- | --- | --- | --- | --- | --- | --- |

Q2. Fühlt sich Ihr Schmerz an wie eingeschnürt oder wie in einem Schraubstock eingeklemmt zu sein?

| kein  Einschnüren | 0 | 1 | 2 | 3 | 4 | 5 | 6 | 7 | 8 | 9 | 10 | schlimmstes vorstellbares Einschnüren |
| --- | --- | --- | --- | --- | --- | --- | --- | --- | --- | --- | --- | --- |

Q3. Fühlt sich Ihr Schmerz wie ein Druck an?

| kein Druck | 0 | 1 | 2 | 3 | 4 | 5 | 6 | 7 | 8 | 9 | 10 | schlimmster vorstellbarer Druck |
| --- | --- | --- | --- | --- | --- | --- | --- | --- | --- | --- | --- | --- |

Q4. Wie lange dauerten Ihre Spontanschmerzen **in den letzten 24 Stunden**?

Kreuzen Sie die Antwort an, die der Dauer am besten entspricht:

dauerhaft (mehr als 12 Stunden) 🞎

zwischen 8 und 12 Stunden 🞎

zwischen 4 und 7 Stunden 🞎

zwischen 1 und 3 Stunden 🞎

weniger als 1 Stunde 🞎

**Schmerzattacken / unterbrochener Schmerz**

Beschreibt eines der beiden Bilder die Schmerzen, wie Sie sie verspüren?

|  | Schmerzattacken (unterbrochener Schmerz) dazwischen schmerzfrei | Schmerzattacken (unterbrochener Schmerz) auch dazwischen Schmerzen |  |
| --- | --- | --- | --- |

Für jede der folgenden Fragen kreuzen Sie bitte die Ziffer an, die **am besten die mittlere Stärke Ihrer Schmerzattacken während der letzten 24 Stunden** angibt. Kreuzen Sie „0“ an, wenn Sie einen solchen Schmerz nicht verspürt haben. (kreuzen Sie bitte immer nur eine Ziffer an)

Q5. Empfinden Sie Ihre Schmerzattacken wie elektrische Schläge?

| überhaupt nicht | 0 | 1 | 2 | 3 | 4 | 5 | 6 | 7 | 8 | 9 | 10 | schlimmste vorstellbare elektrische Schläge |
| --- | --- | --- | --- | --- | --- | --- | --- | --- | --- | --- | --- | --- |

Q6. Fühlt sich Ihr Schmerz stechend an?

| kein Stechen | 0 | 1 | 2 | 3 | 4 | 5 | 6 | 7 | 8 | 9 | 10 | schlimmstes vorstellbares Stechen |
| --- | --- | --- | --- | --- | --- | --- | --- | --- | --- | --- | --- | --- |

Q7. Wie viele dieser Schmerzattacken hatten Sie **in den letzten 24 Stunden**?

Wählen Sie die Antwort, die am ehesten zutrifft:

mehr als 20 🞎

zwischen 11 und 20 🞎

zwischen 6 und 10 🞎

zwischen 1 und 5 🞎

keine Schmerzattacken 🞎

**Schmerzen, die durch bestimmte Auslöser hervorgerufen oder verschlimmert werden**

Haben Sie Schmerzen, die durch bestimmte Auslöser hervorgerufen oder verschlimmert werden, z. B. durch Reiben, Druck, oder Kontakt mit kalten Gegenständen im schmerzhaften Bereich?

Für jede der folgenden Fragen kreuzen Sie bitte die Ziffer an, die am besten der Stärke der Schmerzen, die durch Auslöser hervorgerufenen oder verschlimmerten entspricht, die Sie **im Mittel in den letzten 24 Stunden** hatten. Kreuzen Sie „0“ an, wenn Sie diesen Typ Schmerz nicht verspürt haben. (kreuzen Sie bitte immer nur eine Ziffer an)

Q8. Haben Sie im schmerzhaften Bereich Schmerzen, die durch Reiben hervorgerufen oder verschlimmert werden?

| kein Schmerz | 0 | 1 | 2 | 3 | 4 | 5 | 6 | 7 | 8 | 9 | 10 | maximal vorstellbarer Schmerz |
| --- | --- | --- | --- | --- | --- | --- | --- | --- | --- | --- | --- | --- |

Q9. Haben Sie Schmerzen, die durch Druck auf den schmerzhaften Bereich hervorgerufen werden?

| kein Schmerz | 0 | 1 | 2 | 3 | 4 | 5 | 6 | 7 | 8 | 9 | 10 | maximal vorstellbarer Schmerz |
| --- | --- | --- | --- | --- | --- | --- | --- | --- | --- | --- | --- | --- |

Q10. Haben Sie Schmerzen, die durch Kontakt mit einem kalten Gegenstand im schmerzhaften Bereich hervorgerufen oder verschlimmert werden?

| kein Schmerz | 0 | 1 | 2 | 3 | 4 | 5 | 6 | 7 | 8 | 9 | 10 | maximal vorstellbarer Schmerz |
| --- | --- | --- | --- | --- | --- | --- | --- | --- | --- | --- | --- | --- |

**Gefühlsstörungen**

Haben Sie **im schmerzhaften Bereich** ungewöhnliche Gefühlsstörungen?

Für jede der folgenden Fragen kreuzen Sie bitte die Ziffer an, die **am besten der Stärke Ihrer ungewöhnlichen Gefühlsstörungen** entspricht, die Sie **durchschnittlich in den letzten 24 Stunden** hatten. Kreuzen Sie „0“ an, wenn Sie dieses Gefühl nicht hatten (kreuzen Sie immer nur eine Ziffer an).

Q11. Empfinden Sie ein Kribbeln?

| kein Kribbeln | 0 | 1 | 2 | 3 | 4 | 5 | 6 | 7 | 8 | 9 | 10 | maximal vorstellbares Kribbeln |
| --- | --- | --- | --- | --- | --- | --- | --- | --- | --- | --- | --- | --- |

Q12. Empfinden Sie etwas, das sich anfühlt wie Ameisenlaufen?

| kein Ameisenlaufen | 0 | 1 | 2 | 3 | 4 | 5 | 6 | 7 | 8 | 9 | 10 | maximal vorstellbares Ameisenlaufen |
| --- | --- | --- | --- | --- | --- | --- | --- | --- | --- | --- | --- | --- |

Vielen Dank für Ihre Mitarbeit !
